# Colombian Elders and Their Use of Handheld Digital Devices

**DOI:** 10.3389/fpsyg.2018.02009

**Published:** 2018-11-06

**Authors:** Carmen Ricardo-Barreto, Marco Cervantes, Jorge Valencia, John Cano-Barrios, Jorge Mizuno-Haydar

**Affiliations:** ^1^Department of Education, Universidad del Norte, Colombia, Barranquilla, Colombia; ^2^Department of Psychology, Universidad del Norte, Colombia, Barranquilla, Colombia

**Keywords:** digital divide, internet, elderly adult, digital literacy, mobile phone, information skills

## Abstract

Technological advances in the information and knowledge society have influenced and transformed economic, social, and educational dynamics. Currently there are many digital gaps related to the access to technology, level of digital literacy, and social use. These gaps vary based on the age of the population and become more noticeable among elders. Digital illiteracy leads to the underusing of technological developments of the 21st century, making it difficult to take advantage of all the possibilities that they offer to our society. This study aims to analyze the level of penetration and use of handheld digital devices, especially the cellular phone, among the adult population. This study is based on the 2016 National Survey of Life Quality in Colombia, elaborated by Departamento Administrativo Nacional de Estadística-DANE, specifically on the module of ownership and use of ICT goods. Results of four age groups are compared, which include 32 year olds or under, from ages 33 to 45 years, 46 to 59 years, and over 60 years. The sample consists of 37047 inhabitants of the Colombian territory, grouped by regions (Antioquia, Bogota, Caribbean, Eastern Central, Orinoquia, Amazonia, Pacific, San Andres, and Valle del Cauca). Data have been analyzed by descriptive and inferential statistical procedures, contingency tables analyses, and logistic regression, in order to identify and know the effect of age on the level of penetration and use of handheld digital devices when comparing results in young adults and elderly people. Findings show that young adults use these technologies to a greater proportion when compared to older adults, showing a significant correlation between age and the increase of digital gaps in access and social use of handheld digital devices. Results also make evident that digital divide in Colombia may be associated to the place of residence, as people who live in rural zones are the ones who suffer greater from inequalities.

## Introduction

We all may be aware of the strong presence of technology all around us and how it has become an important element (that is constantly evolving and changing) in our lives. Particularly, the use of Internet and mobile technologies has grown exponentially worldwide. However, the digital gap separating the developed countries from the developing countries is around 43% when measured as a function of the following variables: households with Internet Access, and individuals using the Internet and Mobile broadband subscriptions (Data ICT and Statistics Division, 2015).

This digital divide has become a challenge and a barrier for the entire population, especially for the elderly population because learning how technology works and how to use digital devices implies an outstanding effort (Iancu and Iancu, 2017). According to OECD (2001, p. 5), digital divide refers to “the gap between individuals, households, businesses, and geographic areas at different socio-economic levels with regard to their opportunities to access information and communication technologies (ICTs) and to their use of the Internet for a wide variety of activities.” Olphert et al. (2005) and Warschauer (2003) define digital divide from a democratic point of view that implies differences between those who have access and those who do not, users and non-users of technology in their daily life, and those who are able and those who are unable to take advantage of those technologies.

It is possible to identify many digital gaps related to the opportunities to access ICTs, social use of Internet, and lack of digital literacy. For example, in the last 30 years, the advancement in technology has happened at a rapid pace. Due to this people born prior to 1960 are finding it difficult to adopt new technologies and make them their own as this population was not raised in a society surrounded by digital technologies (Vaportzis et al., 2017).

Digital literacy is achieved when people are able to properly use ICT, and this creates social and economic opportunities for all communities (Federación de enseñanza de Cc. Oo. De Andalucía, 2011). The experience of technology use, especially the Internet, may vary depending on the skills of each user. As new users, it is likely that elders, due to the lack of digital literacy, may have feelings of frustration and inability toward using technology or digital devices (DiMaggio et al., 2001).

In a study carried out among people above 65 years of age, Saracchini et al. (2015) identified that elders showed limitations due to the natural process of aging with regard to the detriment of their listening, vision, and motor skills. They also highlighted the current trend of technological developments, marketing strategies, and service offers toward young people, Cutler (2005), or toward users with technical expertise or digital immigrants (Prensky, 2001). The authors highlight as a very important matter that technologies, characterized by being customizable, modular, and scalable, may be adaptable to the diverse needs of adult population.

Studies in elders carried out in the European Union show that this population is more likely to use traditional mass media than social networks, and they also prefer to use synchronic media such as television, radio, and Newspapers (Unión Europea Fondo Social Europeo, 2010; Nimrod, 2016; Román-García et al., 2016). Such studies agree that the gaps in access, use, and digital literacy between youngsters and elders increase according to age. This is evident in the use of mobiles, computers, and Internet, both in frequency and intensity.

A study performed in USA by Anderson and Perrin (2017), about the use of technologies by elders, shows that a representative percentage of this population has cellular phones, half of which are Smartphones. In the same way, it is evident that the education level is directly related to having and using intelligent phones. Data also show that in this population there are gaps in the access to ICT and in the use of software facilitating social interaction. This may be because elders do not feel sure and comfortable using computers and/or mobile devices. Nevertheless, most of the elders involved in the study have a positive perception of the effect of technology in society. These results are consistent with those obtained by Vroman et al. (2015), who found that most of the adults prefer ICT for family and social communications, and prefer fixed telephony (phone) to mobile technology.

In general, research reveals that mobile devices and access to internet improve life quality at a personal, social, and professional level in elders (Renaud and van Biljon, 2008; Vroman et al., 2015; Öngün and Güder, 2016; Román-García et al., 2016), increase self-esteem (Lustbader, 1997, in Gamberini et al., 2006), and allow the integration to online communities (Iancu and Iancu, 2017). They also provide more access to information (Mikkonen et al., 2002), produce satisfaction, improve self-concept and self-esteem, decrease technological anxiety (Peral et al., 2015), and have a positive impact on the family and society (Aguado et al., 2013).

In the researches we reviewed, some researchers agree that there is a direct relationship between socio-demographic and school level variables, and the access and use of technologies, especially mobile devices (Kwon and Chidambaram, 2000; Renaud and van Biljon, 2008; Unión Europea Fondo Social Europeo, 2010; Vroman et al., 2015; Román-García et al., 2016; Vošner et al., 2016).

This paper analyzes the use of handheld digital devices, especially the cellular phone, in adult population, based on the module of ownership and use of ICT goods of the 2016 National Survey of Life Quality in Colombia, elaborated by el (DANE, 2017). In the 21st century, it is not possible to state that in Colombia there is a real appropriation of ICT because the country is ranked on a basic-low ICT level of Appropriation regarding this matter (Lemoine et al., 2017; Martínez-Coral, 2018). Moreover, there are a few studies about the level of appropriation of these technologies among the older population in Colombia.

In Colombia, technology has had a slow rate of development, especially regarding access and coverage. In the 90’s, the access to mobile technologies was limited, and in 1997, just 3.15% of the population had access to this kind of technology (Fainboim and Rodríguez, 1998). At the beginning of the 21st century, this situation barely changed, reaching only a 5.3% of coverage. In 2010, 40% of the population had access to mobile technology (Orduz, 2011). Regarding the Internet, in 2000, the level of penetration was around 2% of Colombian population (Bustamante and Fajardo, 2001). In 2010, this level increased up to 9.6% (MINTIC, 2011). These data enable us to have an idea of the context in which the target population (elders aged 60 years and above, and born before 1955) lived (Pavez, 2008).

The study aims at identifying the effect of age on the level of penetration and use of handheld digital devices when comparing results in young adults and elderly people by a logistical regression model.

## Materials and Methods

### Design and Measure

The study used a quantitative approach of research with a correlational scope. It is based on data obtained by the National Survey of Life Quality [NSLQ], 2016, elaborated by the Colombian National Statistical Department (Departamento Administrativo Nacional de Estadística, DANE, by its acronym in Spanish) (DANE, 2017). This survey is representative for the entire country. It is a questionnaire that consists of 13 chapters and 299 questions, which provide information about different aspects and dimensions of the welfare of homes including aspects such as access to public, private or community goods and services, health, education, and care of children under 5 years of age.

The analysis units are homes, households and people. The sample consisted of 22893 homes, with a sampling error of 5% in municipal centers^1^ and of 7% in population centers^2^ and rural ones^3^; and with confidence level of 95%. The sample was calculated by a probability, multi-age, stratified and of conglomerate sampling, in which each unit has a known probability, more than zero, of being selected. Given the probabilistic, multi-staged, stratified characteristics of the sampling, the NSLQ had a minimum level of bias (DANE, 2017).

Regarding the information about technologies and communication, the survey aims at measuring the access to these through places of use, frequency of use, activities carried out by Internet, ownership and use of mobile cellular telephone of people of 5 years and above (DANE, 2017). Data were collected between September and November 2016.

### Procedure

For this study, data were obtained from the Information and Communication Technology module which inquires about the frequency of use of computer and Internet, places of access to Internet and services or activities to use Internet and cellular ownership, based on education level, gender, age, and Colombian region, among other aspects, in different Colombian regions as defined by DANE (Antioquia, Bogotá, Caribe, Central, Oriental, Orinoquía – Amazonía, Pacífica, San Andrés, Valle del Cauca).

Based on the NSLQ, processed and validated data were regrouped in two variable sets as follows (DANE, 2017):

(A) Socio-demographics variables
  • Education level
  • Class (residence zone or region)
  • Region
  • Age
  • Gender
(B) Variables about Penetration and Use
  • Cellular phone
  • Conventional cellular phone
  • Smartphone
  • Frequency of use of cellular phone
  • Use: Access to Internet
  • Use: personal or family calls
  • Use: Business calls
  • Use: Text messaging
  • Use: Web browsing

For each subject in the sample, a grade was established based on the following questions selected from NSLQ (DANE, 2017): Do you have cellular phone? Is your cellular phone a Smartphone? Do you use a cellular phone to access the Internet?

As a result, a Proxy variable was consolidated, composed of four categorical levels which account for the level of penetration of mobile devices regarding the access to ICT (Table 1).

**Table 1:** Level of penetration of mobile devices as a function of ownership, use and access of mobile devices.

Level of penetration	Characteristics of the level based on ownership, use, and access to mobile devices
L1: There is no effective incorporation	The subject does not have a mobile device
L2: Technological backwardness	The subject has a mobile device that does not allow access to Internet
L3: Lagging due to lack of knowledge or interest/potential access	The subject has a mobile device which allows access to Internet, although s/he decides not to access
L4: Effective incorporation	The subject has a mobile device which allows access to Internet and s/he does it.

Once the variables were reorganized and there was consistency in the creation of scales, the association level of each variable was evaluated by using the created Proxy variable (dependent) that reflects the level of penetration of mobile devices. To do this, the calculation of frequencies, means and percentage distributions, as well as the JI2 test, Kendall Tau-b and Tau-c, and Spearman’s rho were taken into account.

To interpret these coefficients, Bisquerra (2009) proposes the following orientations for co-relational studies in social sciences, regarding absolute values of the coefficient: (1) Practically null between 0 and 0.20; (2) Low between 0.21 and 0.40; (3) Moderate between 0.41 and 0.70; (4) High between 0.71 and 0.90; and (5) Very High between 0.91 and 1.0. In the same way, it was in contrast to the hypothesis that the coefficient of correlation is significantly different from 0, for an Alpha of at least 0.05.

The inferential analysis consisted of the application of a logistic regression model which accounts for the probability of a citizen to be in L3 and L4 levels (highest levels of penetration) for which the Proxy variable was recoded in a dichotomous version as follows: 0 = Levels of low penetration (L1 and L2), and 1 = Levels of high penetration (L3 and L4). The estimated model is expressed as:

$$P(Y=1)=1/\left(1+\exp^{\left(-\alpha -\beta 1X1-\beta 2X2-\beta 3X3-\cdots \beta nXn\right)}\right)$$

where P (*Y* = 1) is the probability of achieving the category 1; α, β1, β2, β3…, βk are the model parameters, and exp is the simplified exponential function, corresponding to raise the number e to the power contained between parentheses, e being Euler numbers or constant, or the base of Naperian logarithms (its thousandth approximate value is 2.718).

Once the variables with a statistically significant relationship were determined, the regression model was defined. This was carried out in two steps: (1) the evaluation of individual models with each predictor variable in order to value the individual contribution of each one; and (2) the evaluation of the model introducing the variables step by step, evaluating in each step, the distribution of cases classified in as correct way, by determining their specificity and sensitivity. Nominal variables with fictitious codes were used for each category in both the procedures. All statistical procedures were carried out by using SPSS v. 25 software.

## Results

This section has been divided into two parts. The first part presents descriptive and correlational data related to the level of penetration of mobile devices. The second part shows a logistic regression model which informs about the gaps of access, use, and digital literacy of elders in Colombia, based on the survey carried out by Dane (2018).

### Level of Penetration of Mobile Devices

Regarding Colombian regions, it is evident that the highest level of effective penetration of mobile devices (N4) is in Bogota D. C. (Capital city of Colombia). On the other hand, in the Central, Caribbean, and Pacific regions, the level of effective penetration is significantly lower (Table 2). Results described here (Table 3) account for the level of penetration of mobile devices in elders, taking into account the four categories described above in Table 1. It can be observed that 30.3% of Colombians considered in the study achieved the level of effective incorporation, that is, they had the device with access to Internet and used it for accessing social applications on the net, in contrast to 69.7% who had some type of digital divide either because they did not have the suitable device (14.6%) or because they did not have access to Internet (43.8%) or because they did not use it for accessing the net (11.3%).

**Table 2:** Distribution by level of penetration by Region.

Region	Level of Penetration			
	N1	N2	N3	N4
Antioquia	12,5%	42,0%	10,3%	35,2%
Bogotá	3,1%	26,7%	9,7%	60,5%
Caribe	19,5%	51,1%	7,5%	21,9%
Central	12,7%	56,4%	10,8%	20,1%
Oriental	9,1%	48,3%	11,3%	31,2%
Orinoquía – Amazonía	6,0%	42,4%	13,8%	37,8%
Pacífica	26,0%	35,2%	13,3%	25,5%
San Andrés	9,4%	26,8%	10,3%	53,5%
Valle del cauca	13,8%	42,8%	13,1%	30,2%
Total Colombia	14,6%	43,8%	11,3%	30,3%

**Table 3:** Distribution by level of penetration and age range.

	Level of penetration				
Age range	L1	L2	L3	L4
< = 32	11.9%	29.4%	12.5%	46.1%
33 – 45	11.0%	38.1%	12.8%	38.0%
46 – 59	11.9%	50.2%	12.2%	25.7%
60 +	24.4%	58.2%	7.3%	10.2%
Total	14.6%	43.8%	11.3%	30.3%

In the same way, results make evident that the level of effective penetration decreases with age. The younger population has the highest levels of penetration (46.1%), whereas elders over 60 years of age show the lowest levels of penetration (10.2%). Besides, the ownership of mobile devices also decreases with age. A quarter of elders over 60 years do not have mobile devices when compared to younger people, where this proportion is a tenth.

On the other hand, it is important to mention that there is an evident significant association between penetration and age (Kendall Tau-c = −0.245; *p*-value = 0.000). It can be said that the older the subject is, the lesser the participation in higher penetration levels.

According to Spearman’s rho coefficient, there is a statistically significant correlation between the use and frequency of use of cellular phones with subject’s age, gender, and place of residence (Table 4).

**Table 4:** Coefficient of correlation between variables of characteristics of subjects and the uses of mobile devices.

Ítem	Education level	Gender	Zone	Age
Use his/her cellular phone for personal or family calls	−0.169^∗∗^	0.001	0.097^∗∗^	0.115^∗∗^
Use his/her cellular phone for business calls	−0.194^∗∗^	−0.183^∗∗^	0.109^∗∗^	0.175^∗∗^
Use his/her cellular phone for text messages (SMS, instant messaging, chat, and so on)	−0.384^∗∗^	0.005	0.222^∗∗^	0.242^∗∗^
Use his/her cellular phone for navigating the Internet	−0.459^∗∗^	0.016^∗∗^	0.241^∗∗^	0.289^∗∗^
Frequency of use of the cellular phone	−0.262^∗∗^	0.015^∗∗^	0.265^∗∗^	0.162^∗∗^

*^∗∗^Correlation is significant on the level 0.01 (bilateral). ^∗^Correlation is significant on the level 0.05 (bilateral).*

There are low and moderate sinificant correlations between education level and the use of the mobile for accessing to internet and chatting and the frequency of device use. In this case, the higher the education level, the better the usage of devices; and in the case of older people and those living in rural zones, it is found that there is a decrease in the use of navigation and the sending of text messages (Table 4).

### Variables Affecting the Probability of Penetration of Mobile Devices

The logistic regression model is made up of four variables: zone or place of residence, education level, gender, and age, which are independent factors that are predictors of the Level of Penetration. Table 5 shows results of the final model. It includes coefficient of regression (B), the standard error of measurement (ET), and the value of Wald statistic, with degrees of freedom (df), level of significance (Sig), and the relative risk (Exp (B)) of each variable with the respective confidence intervals.

**Table 5:** Results of logistic regression model.

Variable/ Category	*B*	Standard error	Wald	*df*	Sig.	Exp(B)	95% C.I. for EXP(B)	
							Inferior	Superior
Zone (Rural^∗^)	−0.812	0.033	616.999	1	0.000	0.444	0.416	0.473
Education level^∗∗^			3847.855	4	0.000			
Primary	0.623	0.093	45.001	1	0.000	1.865	1.555	2.238
Secondary	1.557	0.095	271,255	1	0.000	4.744	3.942	5.71
High School/T&T	2.255	0.091	610.474	1	0.000	9.539	7.976	11.408
Superior	3.396	0.102	1102.876	1	0.000	29.852	24.43	36.477
Age^∗∗∗^			1383.701	3	0.000			
33 - 45	−0.192	0.033	33.311	1	0.000	0.825	0.773	0.881
46 - 59	−0.679	0.035	374.221	1	0.000	0.507	0.473	0.543
60 +	−1.578	0.045	1205.434	1	0.000	0.206	0.189	0.226
Constant	−1.752	0.092	359.605	1	0.000	0.173		

*^∗^Reference category: Urban Zone. ^∗∗^Reference category: None (without studies). ^∗∗∗^Reference category: < = 32 años. Source: Authors’ elaboration. Data: *N* = 36712.*

The estimation of the “Odds Ratio” Risk [Exp(b)] obtained for each variable provides information about the relation details of each variable to low or high levels of penetration. So, the probability of being in higher levels of penetration in Colombia is as follows:

• 2.56 (1/0.444) times less probable in those surveyed in rural zones.
• 29.852 times more probable among those surveyed with higher education level.
• 4.89 (1/0.206) times less probable among those surveyed over 60 years of age.

The evaluation of the goodness-of-fit of the model was carried out through the deviation (2LL), Cox-Snell’s R2, Nagelkerke’s R2, and Hosmer and Lemeshow’s test. Table 6 shows that Nagelkerke’s R2 coefficient indicates that 33.4% of the variation in the level of penetration may be explained by the model in the ranges accepted in other studies with similar methodologies (Campo-Arias et al., 2005; Rodríguez-Ayán, 2005), with values for these indicators being between 27.5 and 50.4%. Additionally, the percentage of coincidences from the table of classification was 76.4%, which indicates that the model has a good capacity of prediction.

**Table 6:** Adjustment of the Model.

Indicator	Value
−2 log likelihood	35121.654
Cox-Snell’s R2	0.236
Nagelkerke’s R2	0.334
% Correct Category 1	0.761

*Source: Authors’ elaboration.*

## Discussion

In this study, the digital divide regarding penetration level and use of Internet and mobile devices in elders is addressed as a function of three factors that cover a third of the behavior of the level of penetration of mobile devices in Colombia. These are: (1) age, (2) education level, and (3) place of residence, which are part of the socio-demographic profile of subjects. Results sustained the findings of previous research carried out in Europe and United States, and depict the current situation in Colombia, a Latin American developing country.

Results show that there is a negative correlation (Kendall’s Tau-c = −0.245) among the degree of penetration level and use of mobile devices and age. In higher age ranges, low levels of penetration are observed. Similar findings have been found in European countries and in United States (Unión Europea Fondo Social Europeo, 2010; Nimrod, 2016; Román-García et al., 2016; Anderson and Perrin, 2017). Another result of this study is that the use of mobile devices to communicate and access instant messaging and Internet decreases with age, and elders use mobile devices less frequently for social interaction than the younger population. It was also found that there was a relationship between education level and the use of mobile devices. The use of mobile devices increases as the education level improves. Other studies have found similar results (Kwon and Chidambaram, 2000; Renaud and van Biljon, 2008; Unión Europea Fondo Social Europeo, 2010; Vroman et al., 2015; Román-García et al., 2016; Vošner et al., 2016).

The digital divide found in this study may be due to the elders’ difficulty to use technologies as a result of limitations produced by aging, such as impairment of vision, listening and motor skills (Saracchini et al., 2015). Similarly, it is possible that generational limitations play an important role for the population considered as digital immigrants (Prensky, 2001). In this sense, it can be speculated that currently, technological innovations favor young population in terms of usability and navigability without considering that such innovations may not meet elders’ needs, whose requirements would be related to personalized, modular and scalable devices (Saracchini et al., 2015). In summary, as expressed by Anderson and Perrin (2017), elders are neither sure nor comfortable using computers and/or mobile devices, maybe because these devices are not adapted to their needs. Another possible cause of this digital gap could be the low purchasing power of many Colombian elders, considering that results of this study show that most of them do not have mobile devices, or if they do have, those devices do not allow them to access the Internet or have mobile Apps (Vroman et al., 2015; Anderson and Perrin, 2017).

Results also make evident that digital divide in Colombia may be associated to the place of residence, as people who live in rural zones are the ones who suffer greater from inequalities. In Colombian rural zones, the service of Internet and cellular phones is offered with coverage limitations. According to Dane (2018), 65% of people living in urban areas access the Internet using their mobile phones, a percentage that doubles in rural zones. In Colombia, the differences between urban and rural zones are a structural aspect that historically has affected the formulation and actions of public policies. Normally, these are thought and constituted from and for urban zones, leaving the rural zones behind (Pulido et al., 2010).

Furthermore, this lack of access to the Internet and mobile phones is opposed to Colombian legal provisions (Law 1341, July 20th, 2009), which seek for the promotion of the investment in this sector and the development of these technologies, the efficient use of networks, and the promotion of free access without discrimination to the Information Society to all Colombian citizens.

The main limitation of this study is that it confirms some relationships already confirmed in the previous literature. However, Colombian studies about this topic are scarce and this research may become a reference for further studies. This research also provides representative data on the ICT use in Colombia at different ages, because of the large sample of 37047 participants that allows the verification of results.

The results of this study suggested the following research and action lines in order to diminish the digital divide between young people and elders. Firstly, it is required to carry out studies aiming at identifying elders’ perceptions and motivations to use mobile devices, causes of gaps, and the relationship between mobile technology and improvement of quality life of the studied population.

Secondly, public policies should be proposed to promote the access and use of mobile devices in elders, by total or partial benefits that allows them to acquire such devices and to access the Internet. Besides, it is recommended to design and offer programs for awareness and training to favor the development of digital literacy in elders, aiming at mitigating the difficulties in the use and appropriation of technology, which are frequently experimented by digital immigrants.

Finally, it is suggested to promote projects on technological innovation and development, which meet elders’ needs resulting from aging, such as loss of listening, vision and motor skills. Additionally, developing applications and services taking advantages of the emergent technologies that should be designed considering the target population and the diversity of needs presented in this population.

Undoubtedly, the aim should be to train the population in the efficient use of technology in such a way that they use it to live, learn, and work in this changing society with multiple information sources and in particular contexts. In this way, they were trained to solve problems in different life dimensions (Iriarte et al., 2015).

## Data Access

DANE authorizes the use, transformation, and analysis of the information contained in its official web page provided that the source is quoted as (source: National Administrative Department of Statistics: www.dane.gov.co).

The contact information is Colombian National Administrative Department of Statistics –Dane (contacto@dane.gov.co), www.dane.gov.co.

## Confidentiality

Regarding data confidentiality, the data of this study are regulated by Law 79 from 1993 (art. 5), which states that data collected by DANE in censuses and surveys cannot be shared with the wider public, governmental institutions or public authorities, but only in numerical summaries which avoid the possibility of deducing from them any information of individual character. Furthermore, Survey data are open and free access, available at DANE web page. They are anonymized. It is important to highlight that the original data have been modified and transformed in order to guarantee the confidentiality of unit of analysis.

## Ethics Statement

Ethics approval was not required as data employed is extracted from a public repository. Data were obtained from the “National Survey of Life Quality (NSLQ), 2016” carried out by the Colombian National Administrative Department of Statistics (DANE by its acronym in Spanish). DANE authorizes the use, transformation, and analysis of the information contained in its official web page provided that the source is quoted as (source: National Administrative Department of Statistics: www.dane.gov.co).

## Author Contributions

CR-B led the manuscript writing process and theoretical framework construction, co-conceptualized the research study, and participated in data analysis and discussion. MC led the methodological approach, participated in data analysis, and contributed to the writing process. JV led the data analysis, co-led the methodological approach, discussion and contributed to the writing process. JC-B co-conceptualized the research study, co-led the theoretical framework construction, and contributed to the writing process. JM-H translated the article, discussion and contributed to the writing process.

## Conflict of Interest Statement

The authors declare that the research was conducted in the absence of any commercial or financial relationships that could be construed as a potential conflict of interest.

## Footnotes

1 Municipal center: a geographical area defined by an urban perimeter whose limits are established by the Municipal Council (Dane, 2007, p.7).

2 Population center: it is a concentration of a minimum of 20 adjacent housing units in the rural area of a town, which presents urban characteristics such as roads and pedestrian paths (Dane, 2007, p.7).

3 Rural area: it is characterized by sparse location of houses, without layout of road system and lack of public services (Dane, 2007, p.7).
